# Cardiac volumes, function, and arterial stiffness five years after severe preeclampsia: A comparison with sedentary and active controls

**DOI:** 10.1111/cpf.70088

**Published:** 2026-08-24

**Authors:** Camilla Edvinsson, Katarina Steding‐Ehrenborg, Ashwin Venkateshvaran, Lena Erlandsson, Stefan R. Hansson, Erik Hedström

**Affiliations:** ^1^ Obstetrics and Gynecology, Department of Clinical Sciences Lund Lund University Lund Sweden; ^2^ Department of Anesthesia and Intensive Care Helsingborg Hospital Helsingborg Sweden; ^3^ Clinical Physiology, Department of Clinical Sciences Lund Lund University Lund Sweden; ^4^ Department of Clinical Physiology Skåne University Hospital Lund Sweden; ^5^ Department of Obstetrics and Gynecology Skåne University Hospital Lund Sweden; ^6^ Diagnostic Radiology, Department of Clinical Sciences Lund Lund University Lund Sweden; ^7^ Department of Radiology Skåne University Hospital Lund Sweden

**Keywords:** cardiac magnetic resonance imaging, cardiovascular disease, echocardiography, preeclampsia

## Abstract

**Aims:**

Preeclampsia is a pregnancy disorder characterized by hypertension and multiorgan failure related to endothelial damage. It is associated with increased risk of cardiovascular disease later in life, but pathophysiological mechanisms are unclear, and strategies to identify women at risk are missing. The aim was to investigate cardiovascular impact 5 years after severe preeclampsia, compared to normotensive pregnancies.

**Methods:**

The study was powered to detect approximately 5% difference between groups by cardiac magnetic resonance (CMR). Ten women experiencing severe preeclampsia, and 20 women with normotensive pregnancies were included: 10 lifestyle‐matched and 10 physically active. Brachial blood pressure, echocardiography and CMR were performed 4–7 years after pregnancy.

**Results:**

There was no difference in systolic or diastolic blood pressure, and all women were normotensive (103 vs. 106 vs. 108 mmHg, *p* = 0.67; and 66 vs. 66 vs. 64 mmHg, *p* = 0.78). Indexed left ventricular volumes were larger for active controls than for women with prior preeclampsia (98 vs. 84 mL/m^2^, *p* = 0.004; and 41 vs. 32 mL/m^2^, *p* = 0.01). Left ventricular mass, cardiac function and pulse wave velocity did not differ. No participant fulfilled the criteria for diastolic dysfunction.

**Conclusion:**

The difference in left ventricular volumes is most likely related to a physiological adaptation to exercise. The lack of persistent hypertension may be the factor explaining the absence of clinically significant differences between groups using routine investigations for cardiovascular structure and function, despite severe preeclampsia. Structured follow‐up is essential to enable early diagnosis of hypertension after preeclampsia, as well as supporting an active and potentially protective lifestyle.

## 1 INTRODUCTION

Preeclampsia is a pregnancy‐associated hypertensive disorder that affects 3–5% of all pregnancies worldwide. It leads to an increased risk of morbidity and mortality both during pregnancy and postpartum and is, in addition, associated with an increased long‐term risk of cardiovascular disease (Honigberg et al., 2019). However, the cardiovascular effects in pregnancies complicated by preeclampsia are heterogenous (Gyselaers, 2022), and today's improved peripartum management may impact future outcome. Preeclampsia affects the vascular endothelium, leading to arterial stiffness, hypertension and multiorgan failure (Edvinsson et al., 2025; Erlandsson et al., 2023; Ives et al., 2020; Roberts, 1998). Increasing evidence indicates that the vascular changes may also be the primary driver for cardiac disease, where pathological adaptation to hypertension results in long‐term cardiovascular disease (Levine et al., 2022). Although statistically significant differences in left ventricular mass have been shown between women with prior preeclampsia and healthy pregnancies, values are still within the normal range (Boardman et al., 2020) or increased in those with hypertension (Gronningsaeter et al., 2022; Quesada et al., 2020). Furthermore, others have shown that left ventricular hypertrophy present during and immediately after pregnancy disappears within 6 months postpartum, also in women with prior preeclampsia, with normalization of the blood pressure (Kalapotharakos et al., 2020). This means that hypertension as an intermediate driver of left ventricular hypertrophy may be more important than previously believed in women with prior preeclampsia.

Physical activity is central in the prevention of cardiovascular disease (Perry et al., 2023), and a sedentary lifestyle is associated with increased cardiovascular events (Laufs et al., 2005). Physical activity also positively affects placental angiogenic biomarkers during the first half of pregnancy, independent of body mass index (Kozai et al., 2025), and positively affects placental cytokine levels (Acosta‐Manzano et al., 2025). It is unclear whether the severity of preeclampsia reliably predicts the risk of developing cardiovascular disease, and to what extent the severity of complications affects persistent cardiovascular changes. Most importantly, there is still no widely implemented structured clinical routine follow‐up of women after preeclampsia, particularly related to cardiovascular health, and few programmes exist for guiding post‐preeclampsia care (Mosca et al., 2011; Seely et al., 2021).

The aim of this study was therefore to investigate potential cardiovascular changes using echocardiography and CMR approximately 5 years after a pregnancy complicated by severe preeclampsia with need of intensive care, compared to normotensive pregnancies in activity‐matched, that is, sedentary, and active controls.

## 2 METHODS

### 2.1 Ethical permission

The Swedish Ethical Review Authority approved the study, which was performed according to the Helsinki Declaration and complies with the STROBE (STrengthening the Reporting of OBservational studies in Epidemiology) guidelines (von Elm et al., 2008). All participants signed consent before participation.

### 2.2 Study population

This prospective case‐control study included 10 women admitted to intensive care units in the Region of Skåne, Sweden, between the years 2016–2019 due to either early‐ (three patients) or late‐onset (seven patients) severe preeclampsia. Twenty women with healthy normotensive pregnancies who delivered during the same timeframe were included as controls. The controls were divided into two groups: one group matched to a physical activity level similar to that of the women with prior preeclampsia (denoted sedentary controls), and one group with an exercise level well above the WHO criteria of more than 150 min per week (denoted active controls). The reason for physical activity matching was to reduce potential confounding effects of exercise on comparisons between women with prior preeclampsia and healthy controls with normotensive pregnancies. Exclusion criteria for all participants were prior cardiovascular disease, pulmonary disease, smoking within 10 years, diabetes mellitus (gestational or pregestational) or contraindications for CMR.

Preeclampsia was defined according to the ISSHP (the International Society for the Study of Hypertension in Pregnancy) guidelines as *de novo* hypertension after gestation week 20 associated with maternal organ involvement and/or foetal growth restriction (Brown et al., 2018). Haemolysis, elevated liver enzymes, low platelets (HELLP) syndrome and eclampsia were considered as serious manifestations of preeclampsia and not as separate disorders (Brown et al., 2018). Severe preeclampsia was defined as systolic blood pressure > 160 mmHg and/or diastolic blood pressure > 110 mmHg (Tranquilli et al., 2013).

Examinations for the current study included echocardiography and blood pressure measurements in conjunction with CMR.

### 2.3 Blood pressure measurements

Blood pressure measurements were acquired in the supine position using an oscillometric device with an appropriately sized automated cuff around the right arm (Invivo, Philips Medical Systems, validated as for routine clinical use). The brachial arterial systolic and diastolic pressure were measured once before the CMR examination begun and twice during the CMR examination. The two latter blood pressures were averaged and reported in this publication to reflect a resting blood pressure.

### 2.4 Echocardiography

Echocardiography primarily served as a measure to control for the presence of diastolic dysfunction. It was performed by a credentialed sonographer (A.V.; over 20 years' experience) using a Vivid E95 ultrasound system (GE Ultrasound, Horten, Norway) according to current recommendations (Caballero et al., 2015; Lang et al., 2015), including evaluation of diastolic dysfunction (Nagueh et al., 2016). Images were stored and analyzed offline in EchoPac 11.0.0.0 (GE Ultrasound, Waukesha, Wisconsin). To reduce potential bias, all analyses were performed by the same sonographer in a blinded randomized order after completion of data acquisition. Mitral inflow was measured using pulsed‐wave Doppler in the apical four‐chamber view. Trans‐mitral early (E) and late (A) diastolic velocities were obtained, and the mitral E/A ratio was calculated. Mitral deceleration time was calculated as the time from maximum flow velocity over the valve until zero velocity.

Early mitral annular velocities (e') were measured at the septal and lateral walls using Doppler tissue imaging and the mean E/e' ratio was calculated. Global longitudinal strain was assessed using two‐dimensional speckle tracking and calculated as the mean of the global peak systolic strain from each of the three apical two‐, three‐ and four‐chamber views.

### 2.5 Cardiac magnetic resonance imaging

Participants were imaged in the supine position using a 1.5T CMR scanner (Aera, Siemens Healthineers) with a combination of a spine coil and a cardiac array coil with retrospective ECG (electrocardiogram) gating. Balanced steady‐state free precession cine images were acquired in end‐expiration in the standard short‐axis and 4‐, 3‐, and 2‐chamber views for measurements of volumes and function. For thoracic aortic pulse wave velocity, 2D phase‐contrast flow with more than 35 timeframes over the cardiac cycle was acquired in free breathing in the ascending aorta and in the descending aorta at the level of the diaphragm, together with a white‐blood 3D isotropic T2‐prepared sequence for centerline distance.

Images were analyzed using the software Segment 4.0 R13109 (Medviso) (Heiberg et al., 2010) with observers blinded to all other data. Left endocardial and epicardial borders, and right endocardial borders were manually delineated in short‐axis images at end diastole and end systole for ventricular volumes and left ventricular mass (Hedström et al., 2017). Long‐axis images were used for guidance. Ventricular volumes and function, and left ventricular mass were measured in consensus by two observers (K.S.E. and E.H. with 20 and 25 years' CMR experience, respectively). Stroke volume was calculated as end‐diastolic volume minus end‐systolic volume, and ejection fraction was calculated as stroke volume divided by end‐diastolic volume and expressed in percent. Left ventricular mass was calculated as left ventricular wall volume (without papillaries) times myocardial density (1.05 g/mL). To account for variations in body size, all variables except ejection fraction were indexed to body surface area.

Pulse‐wave velocity was measured by E.H. Aortic centerline distance was measured manually in the 3D white‐blood images and defined as the distance between the flow planes in the ascending aorta and at diaphragm level. The flow curves were obtained by automatic vessel delineation (Bidhult et al., 2019), with manual correction if needed. Pulse wave velocity was assessed using the time‐to‐foot approach, including baseline correction (Dorniak et al., 2016; Lundström et al., 2022).

### 2.6 Statistical analysis

Statistical analyses were performed in GraphPad Prism 10.4.1.532 (GraphPad software). Continuous variables were expressed as median and interquartile range, after normality was dismissed using histograms and the skewness of data. The number of participants was chosen to detect differences between groups by CMR. It was based on sample size calculations by Bellenger et al (Bellenger et al., 2000), which considers the high precision of CMR, combined with experience from CMR measurements after pregnancy complicated by preeclampsia (Kalapotharakos et al., 2020) and previously published differences between such groups (Boardman et al., 2020; Gronningsaeter et al., 2022; Hedström et al., 2017; Quesada et al., 2020). The study was thus not powered to detect differences by echocardiography; however, statistical tests were performed for completeness with this in mind. The Kruskal–Wallis test assessed differences between groups with Dunn's correction for multiple comparisons with multiplicity‐adjusted *p* values reported in case of statistical significance and in graphs, whereas groupwise *p* values otherwise are reported. *p* values < 0.05 were considered to show significant differences.

## 3 RESULTS

### 3.1 Study population

Table 1 describes the women with prior preeclampsia at the time of pregnancy. The reasons for admission to the intensive care unit were HELLP, eclampsia or severely dysregulated blood pressure that could not be managed at the delivery ward. The median stay at the intensive care unit was 1 day (range 1–3 days). Both systolic and diastolic blood pressures were elevated, as per the definition of preeclampsia at the time of delivery. Women with preeclampsia were more often nulliparous (0 (0–1)) before the index pregnancy compared to the sedentary controls (1 (0–3); *p* = 0.02) but not as compared to the active controls (1 (0–2); *p* = 0.23). Furthermore, women with prior preeclampsia did not become pregnant again after their index pregnancy. This means that the otherwise additional risk of recurrent preeclampsia did not affect the results in the current study.

**TABLE 1:** Maternal and foetal characteristics at time of delivery.

	Patients	Sedentary controls	Active controls	*p*
Age (years)	33 [26–40]	34 [31–37]	31 [30–37]	0.78
Maternal body mass index (kg/m^2^)	25.5 [20.0–32.0]	30.0 [25.4–32.8]	25.7 [24.1–29.4]	0.37
Gestational age at delivery (days)	250 [228–261]	284 [276–291]	281 [270–285]	**<0.001** ^**#**^
Birthweight (g)	2153 [1515–2447]	3779 [3403–4079]	3376 [3060–3935]	**<0.001** ^**§**^
Highest systolic blood pressure (mmHg)	166 [155–173]	116 [107–123]	N/A	**<0.001**
Highest diastolic blood pressure (mmHg)	108 [91–123]	75 [66–78]	N/A	**<0.001**

*Note*: Values are median [interquartile range]. ^#^ *p* < 0.001 between preeclampsia and sedentary controls, and *p* = 0.01 between preeclampsia and active controls. ^§^ *p* < 0.001 between preeclampsia and sedentary controls, and *p* < 0.01 between preeclampsia and active controls. There were no differences between sedentary and active controls.

Table 2 shows maternal characteristics at the time of the current study. One woman from the active controls group was excluded due to sequelae to pneumonia discovered after inclusion. There were no differences in systolic or diastolic blood pressure between groups. One of the women with prior preeclampsia was on medication for hypertension, with a combination of a calcium‐blocker and an angiotensin‐converting enzyme (ACE) inhibitor, resulting in normotension (129/81 mmHg).

**TABLE 2:** Maternal characteristics at the time of current study.

	Patients (*n* = 10)	Sedentary controls (*n* = 10)	Active controls (*n* = 9)	*p*
Time since delivery (years)	5.5 [4.8–7.0]	6.0 [5.0–6.3]	5.3 [4.4–6.9]	0.69
Age (years)	38 [32–47]	40 [37–44]	36 [34–42]	0.56
Maternal height (cm)	164 [159–167]	165 [163–171]	175 [172–181]	**0.01** ^**#**^
Maternal weight (kg)	67 [61–79]	68 [59–85]	63 [61–79]	0.97
Maternal body mass index (kg/m^2^)	25.1 [22.6–30.2]	25.0 [22.2 – 30.3]	21.1 [20.3–25.1]	0.13
Systolic blood pressure (mmHg)	103 [98–111]	106 [98–113]	108 [105–112]	0.67
Diastolic blood pressure (mmHg)	66 [57–73]	66 [55–70]	64 [59–66]	0.78

*Note*: Values are median [interquartile range]. ^#^ *p* = 0.01 between preeclampsia and active controls, *p* > 0.06 for the other comparisons.

### 3.2 Echocardiography

**FIGURE 1:**
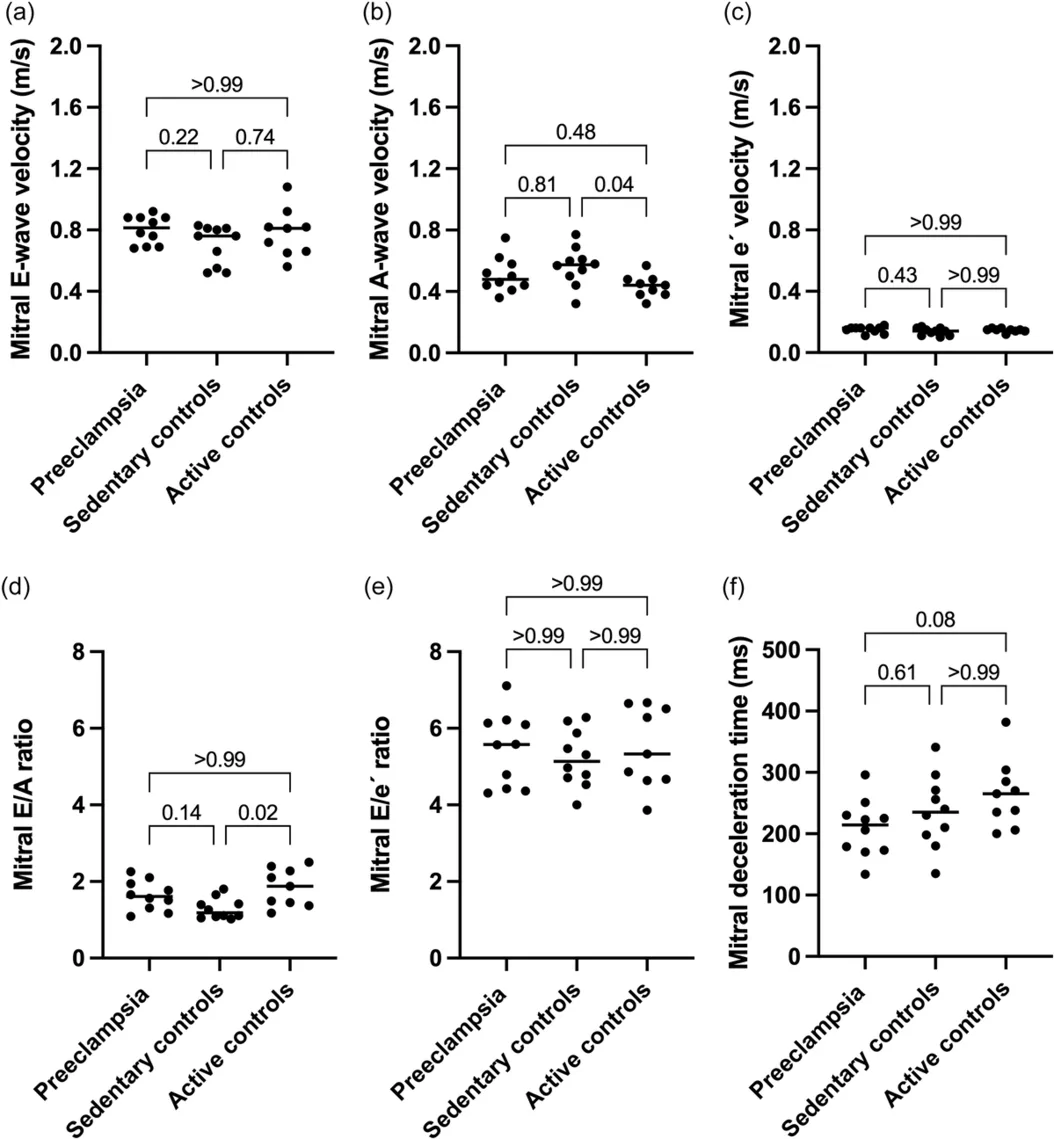
Measurements by echocardiography. Mitral E‐wave velocity (a), mitral A‐wave velocity (b), mitral e´ velocity (c), mitral E/A ratio (d), mitral E/e´ ratio (e), and mitral deceleration time (f). There were no differences between women with prior preeclampsia and the two control groups, respectively. No participant fulfilled clinical criteria for diastolic dysfunction.

Table 3 and Figure 1 show echocardiography data. Velocities over the mitral valve did not differ between women with prior preeclampsia and sedentary controls, either during early filling or during atrial contraction. Active controls, however, had lower velocities over the mitral valve during atrial contraction compared to sedentary controls (0.44 ms vs. 0.58 ms, *p* = 0.04). There was no difference in E/A ratio (peak velocity blood flow during early filling vs. peak velocity flow during atrial contraction) between women with prior preeclampsia and sedentary controls. However, the E/A ratio was higher in active controls compared to sedentary controls (1.9 vs. 1.2, *p* = 0.02). Two women with prior preeclampsia had an E/A ratio > 2 (2.1 and 2.3). Both had a normal E/e´ ratio < 8, whereas one of them had a mitral deceleration time of > 240 ms (296 ms). Also, of the active controls, four had an E/A ratio > 2 (2.1, 2.3, 2.4, and 2.5). Of these, all had a normal E/e´ ratio, and one had a mitral deceleration time of 382 ms. Further, in the same group of active controls, another four had a mitral deceleration time >240 ms (265, 270, 285, and 304 ms). Thus, no participant fulfilled criteria for diastolic dysfunction.

**TABLE 3:** Maternal echo and CMR measurements at the time of current study.

	Patients (*n* = 10)	Sedentary controls (*n* = 10)	Active controls (*n* = 9)	*p*
Echocardiography				
Global longitudinal strain (%)	−19 [−17 to −20]	−18 [−17 to −21]	−20 [−19 to −21]	0.09
Mitral E‐wave velocity (m/s)	0.82 [0.69– 0.88]	0.76 [0.54–0.81]	0.81 [0.66–0.88]	0.19
Mitral A‐wave velocity (m/s)	0.48 [0.43– 0.59]	0.58 [0.49–0.63]	0.44 [0.38–0.48]	**0.04** ^**#**^
Mitral E/A ratio	1.6 [1.3– 2.0]	1.2 [1.1–1.5]	1.9 [1.4–2.3]	**0.02** ^**§**^
Mitral e´ velocity (m/s)	0.16 [0.14– 0.16]	0.14 [0.11–0.16]	0.15 [0.14–0.16]	0.34
E/e´ ratio	5.6 [4.4– 6.2]	5.1 [4.7–6.0]	5.3 [4.7–6.6]	0.76
Mitral deceleration time (ms)	215 [172– 235]	235 [194–277]	265 [221–295]	0.09
Cardiovascular magnetic resonance imaging				
Left ventricular end‐diastolic volume indexed to BSA (mL/m^2^)	84 [78–88]	88 [76–96]	98 [93–110]	**0.004** ^**†**^
Left ventricular end‐systolic volume indexed to BSA (mL/m^2^)	32 [29–37]	37 [32–41]	41 [39–45]	**0.01** ^**°**^
Left ventricular ejection fraction (%)	60 [58–62]	57 [55–60]	59 [57–61]	0.28
Cardiac index (L/min/m^2^)	3.2 [3.0–3.6]	3.0 [2.8–3.6]	3.2 [3.0–3.7]	0.54
Left ventricular mass indexed to BSA (g/m^2^)	43 [37–45]	44 [41–48]	44 [41–47]	0.30
Right ventricular end‐diastolic volume indexed to BSA (mL/m^2^)	84 [80–91]	86 [77–102]	103 [90–114]	0.05^a^
Right ventricular end‐systolic volume indexed to BSA (mL/m^2^)	36 [32–40]	37 [33–49]	44 [36–48]	0.31
Right ventricular ejection fraction (%)	58 [54–61]	53 [51–62]	59 [54–63]	0.51
Thoracic aortic pulse wave velocity (m/s)	4.8 [4.5–5.5]	5.0 [4.2–5.8]	5.2 [4.2–5.5]	0.97

*Note*: Values are median [interquartile range]. BSA, body surface area. ^#^ *p* = 0.04 between sedentary and active controls, *p* > 0.48 for the other comparisons. ^§^ *p* = 0.02 between sedentary and active controls, *p* > 0.14 for the other comparisons. ^†^ *p* = 0.004 between preeclampsia and active controls, *p* > 0.05 for the other comparisons. ^°^ *p* = 0.01 between preeclampsia and active controls, *p* > 0.21 for the other comparisons. ^a^ *p* = 0.05 between preeclampsia and active controls, *p* > 0.15 for the other comparisons.

### 3.3 Cardiac magnetic resonance imaging

**FIGURE 2:**
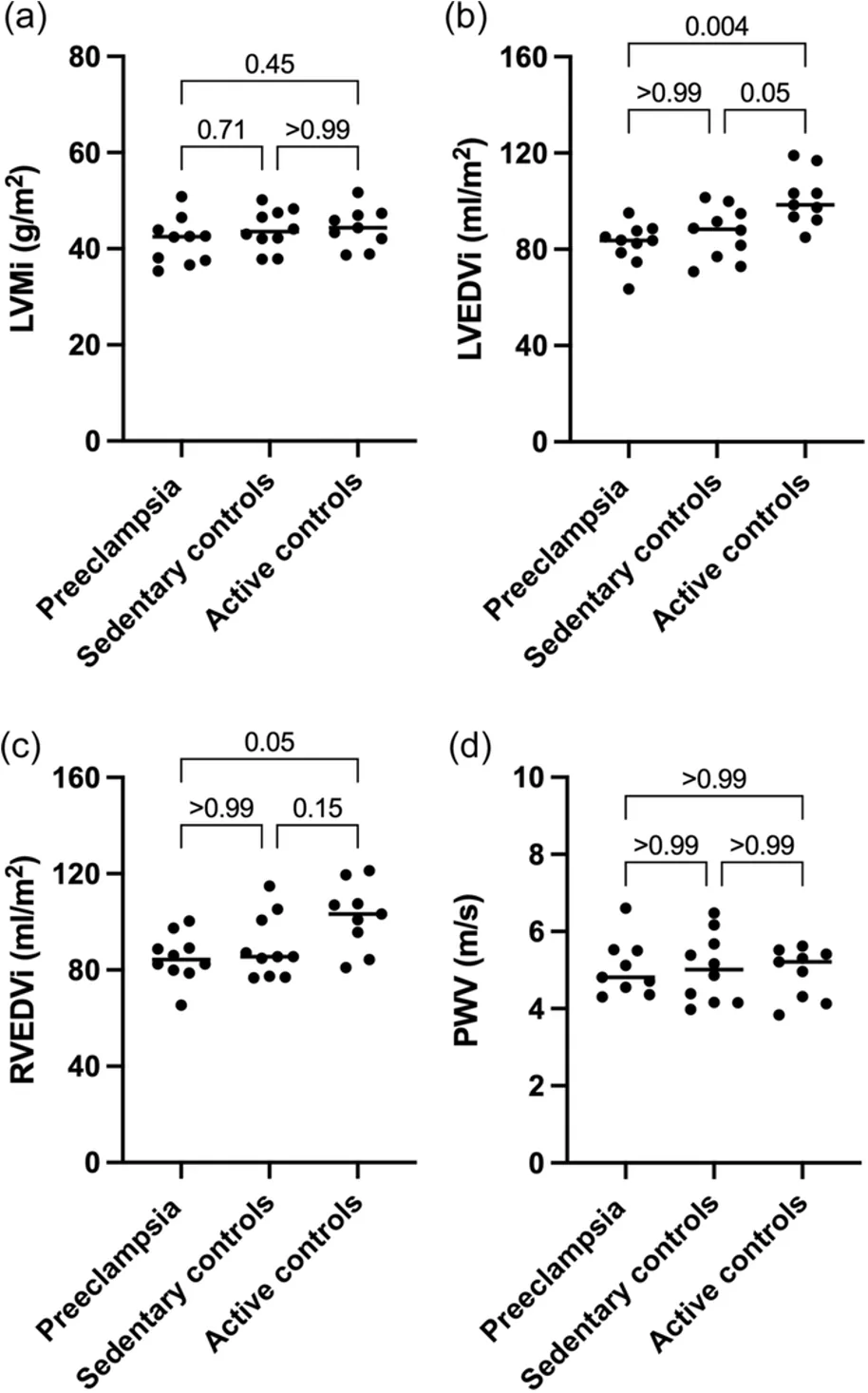
Measurements by cardiovascular magnetic resonance imaging. Left ventricular mass indexed to body surface area (a; LVMi), left ventricular end‐diastolic volume indexed to body surface area (b; LVEDVi), right ventricular end‐diastolic volume indexed to body surface area (c; RVEDVi), and thoracic aortic pulse wave velocity (d; PWV). Active controls had larger, or tendencies towards larger, volumes, which is physiologically expected. There was no increased left ventricular mass in women with prior preeclampsia in this population with normotension, which is in line with previous studies including normotensive versus hypertensive participants (c.f. Discussion).

Table 3 and Figure 2 show volumes and function as obtained by CMR. For pulse‐wave velocity, one woman with prior preeclampsia was excluded due to a large variation in heart rate during the two flow acquisitions. The active controls had larger indexed left ventricular end‐diastolic and end‐systolic volumes than women with prior preeclampsia (98 mL/m^2^ vs. 84 mL/m^2^, *p* = 0.004; and 41 mL/m^2^ vs. 32 mL/m^2^, *p* = 0.01). There were no differences in left ventricular mass, cardiac index, ejection fraction or thoracic pulse wave velocity between the groups.

## 4 DISCUSSION

This study shows no differences in cardiac volumes, function or left ventricular mass between women 4–7 years after severe preeclampsia with need of intensive care compared to after normotensive pregnancy in women with a sedentary lifestyle. As the included women with prior preeclampsia can be seen as being severely ill at time of delivery and could be expected to show pathological cardiac adaptation, the results challenge the notion that women with prior preeclampsia have increased left ventricular mass and impact on cardiac function by preeclampsia on its own. Women with an active lifestyle showed larger indexed left ventricular end‐diastolic and end‐systolic volumes, as can be expected (Bramell et al., 2025).

Even though the E/A ratio was higher in both women with prior preeclampsia and active controls compared to sedentary controls, no participant fulfilled clinical criteria for diastolic dysfunction. The findings of a higher E/A ratio in women with a history of severe preeclampsia are in line with the increased E/A ratio that can be seen in the first trimester in women with recurrent preeclampsia (Sep et al., 2011). An increased E‐wave velocity together with a decreased A‐wave velocity can be found when ventricular relaxation is impaired and left ventricular end‐diastolic filling pressures are increased. During a pregnancy complicated by preeclampsia, this may be explained by an increased left ventricular stiffness (Sep et al., 2011). It is known that the E/A ratio should be cautiously interpretated in relation to the other measurements for restrictive filling, i.e., the E/e´ ratio and mitral deceleration time (Nagueh et al., 2016). These were not fulfilled to qualify as markers of diastolic dysfunction in any participant. Further, the E/A ratio should not be used as an independent marker of diastolic function, particularly when the ejection fraction is preserved, as was the case for the included participants. However, markers of diastolic dysfunction may otherwise be of particular interest after preeclampsia as a biophysical parameter to discover cardiac dysfunction early, when the patients are still asymptomatic, as well as to improve long‐term outcome. This is particularly important considering that heart failure with preserved systolic function is more common in women compared to men (Brandt et al., 2022; Civieri et al., 2023). For the active controls, on the other hand, an increased E/A ratio could be related to the reduced A‐wave velocity, suggesting a lower contribution of atrial contraction to left ventricular filling due to increased preload (Janik et al., 2025).

There were no differences in pulse wave velocity between the groups. This is likely related to a lack of blood pressure differences between groups, as hypertension otherwise is related to increased arterial stiffness (Boutouyrie et al., 2021). It may be that the included women with prior preeclampsia had a lower cardiovascular risk profile except for their severe preeclampsia, and that hypertension may occur later. Thus, continued assessment of blood pressure over time, also late after preeclampsia may be important as persistent hypertension has been linked to early vascular aging reflected as increased aortic stiffness measured by pulse wave velocity (Werlang et al., 2023), with more pronounced aortic stiffness in patients with persistent hypertension compared to women with normalized blood pressure (Paquin et al., 2024).

As the current study only included well‐characterized women with a history of severe preeclampsia, it could be expected that these women would exhibit increased left ventricular mass, as discussed in prior studies (Boardman et al., 2020; Gronningsaeter et al., 2022; Hauge et al., 2024; Quesada et al., 2020). However, this was not the case. Furthermore, left ventricular mass did not differ between groups, and all included women had normal left ventricular mass considering age and sex‐dependent reference ranges. This is in contrast to the results by Boardman et al (Boardman et al., 2020). However, although Boardman et al (Boardman et al., 2020). showed statistically significant differences for left ventricular mass between women with prior preeclampsia and healthy controls (50 [45–55] vs. 46 [40–52] g/m^2^), a majority of their participants, including those after preeclampsia had normal indexed left ventricular mass as based on their reported statistical distribution. Thus, although left ventricular mass was statistically significantly different on a group level, likely related to the large sample size, the importance for the individual patient and thus the clinical significance of left ventricular mass measurements remains unclear. Further, the findings of a relatively higher left ventricular mass in women with prior preeclampsia, as seen by Gronningsaeter et al. (2022) and Quesada et al. (2020), were associated with hypertension. The current study's lack of differences in left ventricular mass between women with prior preeclampsia and controls may thus relate to the lack of persistent hypertension. This is further supported by that left ventricular mass normalizes within 6 months post‐partum after severe late‐onset preeclampsia, correlating to a simultaneous normalization of blood pressure (Kalapotharakos et al., 2020). A recent study by Hauge et al (Hauge et al., 2024). using computed tomography showed a higher prevalence of left ventricular hypertrophy in women with prior preeclampsia 0–28 years ago compared to controls (14 vs. 6%). This may at least in part be explained by that several of the included women had chronic hypertension (41% and 31%) and dyslipidemia (48% and 44%). For comparison, only one of the participants in the current study had medication against hypertension with normalized blood pressure, and none had left ventricular hypertrophy. Even though left ventricular hypertrophy was statistically different between groups in the study by Hauge et al., indexed left ventricular mass overlapped between the women with prior preeclampsia and controls (53 [48–60] g/m^2^ and 52 [47–58] g/m^2^). Taking the results from these research studies together, it should be noted that it is challenging to draw clinical conclusions based on left ventricular hypertrophy in the individual patient. This means that other measurements beyond that of clinical routine echocardiography and cardiac magnetic resonance imaging will be needed for the determination of future cardiovascular risk after preeclampsia.

The chosen follow‐up time after delivery in the current study was based on a meta‐analysis showing that the risk for cardiovascular disease is greatest during the first 10 years after pregnancy (Wu et al., 2017). Even though the current study is powered to detect an approximately 5% difference in variables between groups by CMR, which could be deemed clinically important for the individual patient, the absence of statistically significant differences could be due to the sample size. It is, however, more likely that the lack of statistically significant differences is dependent on physiology, in this case related to the absence of persistent hypertension in those with prior preeclampsia despite that they had severe disease during pregnancy. This is also corroborated by the overlap between groups and differences found between women with prior preeclampsia and active controls.

The current study provides novelty in matching controls to the level of exercise in the group of women with prior preeclampsia. Prior studies do not take physical activity into consideration (Boardman et al., 2020; Gronningsaeter et al., 2022; Quesada et al., 2020), which makes direct comparisons challenging. The matched exercise level may in part contribute to the lack of differences between women with prior preeclampsia and sedentary controls. This was by design, and the effects of exercise are supported by that differences were found between active controls and the other groups. Moreover, the current study included well‐characterized women with prior preeclampsia who only had preeclampsia once. This avoided the risk of the impact of recurrent disease that otherwise would add an extra strain on the cardiovascular system (Auger et al., 2017). However, as prior studies have not always been specific about repeat pregnancy, it also makes direct comparisons between studies challenging.

In summary, the results show generally normal values and normotension in women after severe preeclampsia in need of intensive care. This indicates that the increased risk for cardiovascular disease shown in other studies after preeclampsia may be driven primarily by persistent hypertension, as also discussed by Kalapotharakos et al. (2020) and Levine et al. (2022) and/or a sedentary lifestyle (Kitt et al., 2024).

### 4.1 Limitations

The sample size was dimensioned for CMR and not for echocardiography, why statistically significant differences in ultrasound parameters could have been missed because of a small sample size, that is, introducing type II errors. However, a strength regarding the echocardiography examinations was that all examinations were performed and analyzed by the same experienced sonographer. Also, if anything, the hearts of women with prior preeclampsia showed a tendency towards smaller left ventricular end‐diastolic volume and mass. This also speaks against that a potential left ventricular hypertrophy in the women with prior preeclampsia was missed. The level of physical activity was estimated by the participants themselves, which is a known weakness compared to objective measures. Groups were not matched for parity. Although cardiac remodelling also occurs during normal pregnancy, it is reversible (Ferreira et al., 2021). For preeclampsia, however, remodelling may persist longer, and parity is therefore more important in this group. Since none of the patients with prior preeclampsia was pregnant more than once, this potential impact on the cardiovascular system was eliminated.

Twenty‐four‐hour ambulatory blood pressure could have added additional information about blood pressure, concretely to be able to detect masked hypertension. However, this was not part of the current study.

The different follow‐up times, 4–7 years postpartum, may have influenced the presence of cardiovascular changes. However, there was no obvious visual trend of changes related to increasing time after delivery.

## 5 SUMMARY AND CONCLUSION

This study used echocardiography and CMR to investigate cardiovascular changes 4–7 years after a pregnancy complicated by severe preeclampsia leading to intensive care, and showed no major differences compared to after normotensive pregnancies.

The lack of persistent hypertension in the current study may be the protective factor explaining the lack of differences between groups. This points to the importance of introducing structured and standardized follow‐up programmes after pregnancy complicated by preeclampsia for early detection of hypertension and to encourage all women to perform regular exercise according to recommendations from the WHO.

## AUTHOR CONTRIBUTIONS

**Camilla Edvinsson, Stefan R. Hansson and Erik Hedström:** Conceptualization. **Katarina Steding‐Ehrenborg, Ashwin Venkateshvaran and Erik Hedström:** Methodology. **Camilla Edvinsson, Katarina Steding‐Ehrenborg, Ashwin Venkateshvaran and Erik Hedström:** Formal analysis. **Camilla Edvinsson, Katarina Steding‐Ehrenborg, Ashwin Venkateshvaran and Erik Hedström:** Investigation. **Camilla Edvinsson, Lena Erlandsson, Stefan R. Hansson and Erik Hedström:** Resources. **Camilla Edvinsson, Katarina Steding‐Ehrenborg, Ashwin Venkateshvaran and Erik Hedström:** Data curation. **Camilla Edvinsson:** Writing—original draft preparation. **Camilla Edvinsson, Katarina Steding‐Ehrenborg, Ashwin Venkateshvaran, Lena Erlandsson, Stefan R. Hansson and Erik Hedström:** Writing—review and editing. **Camilla Edvinsson and Erik Hedström:** Visualization. **Stefan R. Hansson, Lena Erlandsson and Erik Hedström:** Supervision. **Camilla Edvinsson, Stefan R. Hansson and Erik Hedström:** Project administration.

## CONFLICT OF INTEREST STATEMENT

The authors declare no conflicts of interest.

## Data Availability

The data that support the findings of this study are available on request from the corresponding author. The data are not publicly available due to privacy or ethical restrictions.
